# *ANATOMIADI*: a national, image-based anatomy competition for residents in Italy to enhance postgraduate education through interactive learning

**DOI:** 10.1186/s13244-026-02389-z

**Published:** 2026-08-29

**Authors:** Carlo Cosimo Quattrocchi, Marinella Neri, Monica Antolini, Alessio Apollonio, Andrea Casula, Flora De Rosa, Veronica Domestico, Marianna Fusina, Paolo Giorgini, Giuseppe Greco, Daniel Raimi, Fabrizio Sannino, Daniele Vo, Andrea Falzone

**Affiliations:** 1 Centre for Medical Sciences, University of Trento, Trento, Italy; 2 Department of Radiological Sciences and Medical Imaging, ASUIT, Rovereto (Trento), Italy; 3 RJC Soft S.r.l., Pisa, Italy; 4 Department of Information Engineering and Computer Science, University of Trento, Trento, Italy

**Keywords:** Diagnostic imaging, Education, Learning, Teaching, Gamification

## Abstract

**Background:**

For radiology residents, the steep learning curve of cross-sectional anatomy (especially in CT and MRI) demands more efficient and engaging educational tools. In recent years, game-based learning (GBL) and gamification have emerged as powerful strategies to enhance postgraduate education by leveraging ‘active recall’ and ‘spaced repetition’ within a competitive framework. Here we describe the context of *ANATOMIADI* and assess the perceived educational impact.

**Materials and methods:**

Image datasets were anonymized, archived, and manually labeled to highlight specific anatomical structures. Over 400 unique games were developed, categorized by anatomical system and tiered into three levels of difficulty. A structured feedback survey was designed and distributed to the participants after the event to capture data on different domains regarding the educational value, gamification and engagement, platform usability and team collaboration, technical workflow, logistics, scientific accuracy, fairness, and quality assessment.

**Results:**

*ANATOMIADI* challenge registered an attendance of 141 participants representing 28 residency programs across Italy. The evaluation of the event’s perceived value yielded a unanimous positive response; indeed, 100% of the participants recommended that the competition be repeated in future academic years. Based on the required level of knowledge, the event was recommended to residents in their 3rd (54/63 responses) and 4th year (48/63 responses) of postgraduate education.

**Conclusion:**

By integrating high-fidelity imaging with interactive game mechanics, the *ANATOMIADI* challenge not only fostered anatomical learning but also built the foundation for a more collaborative and technologically adept generation of radiologists.

**Key Points:**

- **Question:** In the era of high-resolution multidetector CT and advanced MRI, the volume of anatomical data that a resident in radiology must process is unprecedented.
- **Findings:** We developed a context using image-based puzzle games to challenge different residency schools in radiology on imaging-based anatomical structures and rules.
- **Critical relevance statement:** *ANATOMIADI* is the first national competition based on radiological anatomy and dedicated to residents. By using an inclusive “twinning” framework among residency programs, it transforms dense theoretical study into a highly motivating, collaborative, and social learning experience.

**Graphical Abstract:**

**Figure:**
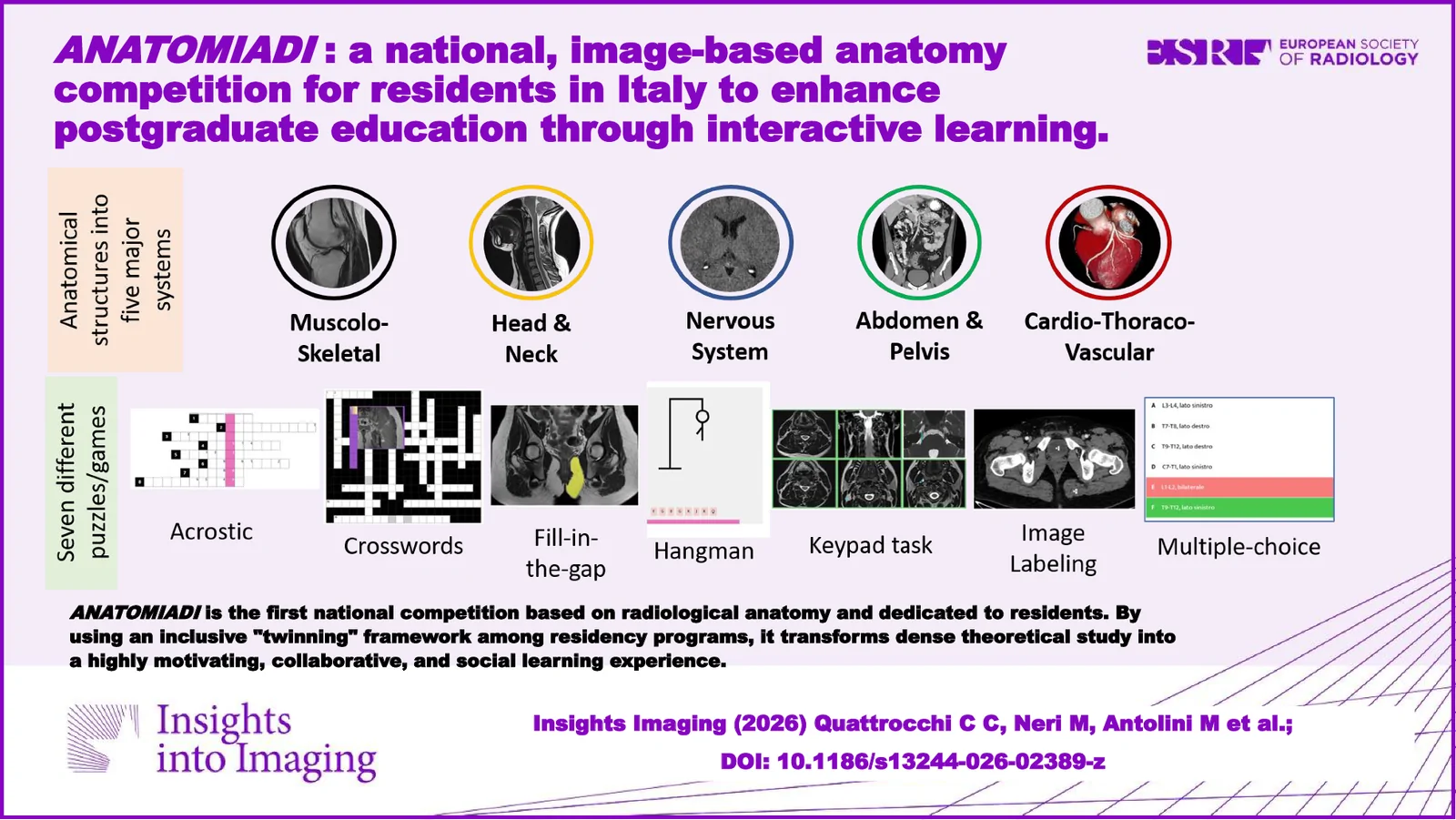

## Introduction

Anatomy represents the fundamental cornerstone of diagnostic imaging and clinical practice in radiology. Traditional teaching models, often based on passive memorization of 2D atlases, frequently fail to bridge the gap between theoretical knowledge and the complex 3D interpretative skills required in a modern clinical setting [1]. The mastery of radiological anatomy is, indeed, no longer a static exercise in memorization but a dynamic process of spatial synthesis. In the era of high-resolution multidetector CT and advanced MRI, the volume of anatomical data that a resident must process is unprecedented. Traditional pedagogical models often struggle to keep pace with this technological evolution, leading to a gap between theoretical knowledge and clinical diagnostic performance [2–4].

For radiology residents, the steep learning curve of cross-sectional anatomy (especially in CT and MRI) demands more efficient and engaging educational tools. In recent years, game-based learning (GBL) and gamification have emerged as powerful strategies to enhance postgraduate education by leveraging ‘active recall’ and ‘spaced repetition’ within a competitive framework [5–8]. By integrating competitive elements and interactive feedback, these methods can significantly increase trainee engagement, enhance long-term retention, and accelerate the development of visual pattern recognition.

Within this framework, we developed the idea of a context using image-based puzzle games to challenge different radiology residency schools in Italy on the identification of anatomical structures and knowledge of anatomical rules. As such, we coined the term “*ANATOMIADI*” to recall the spirit of the Olympic Games (Olimpiadi in Italian language) and refer to a specific competitive yet collaborative environment aiming at promoting a faster, more intuitive mastery of radiological anatomy. The rationale behind this program is that the synergy between educational psychology and advanced visualization technology not only streamlines the learning curve in postgraduate students but also fosters a standardized, high-level competency across residency schools, ultimately improving diagnostic confidence in future radiologists.

The objective of this study is to describe and explain the context of *ANATOMIADI* and assess the educational impact and perceived effectiveness of the first edition of the competition by analyzing the results of the post-event feedback survey. Specifically, we aim to assess the perspectives of participants (residents, expert radiologists playing as referees in the games, and academic staff) regarding the impact of *ANATOMIADI* on knowledge, its pedagogical structure, and the role of integrating gamified technology in postgraduate radiology training.

## Materials and methods

### Image database and tagging

**Fig. 1:**
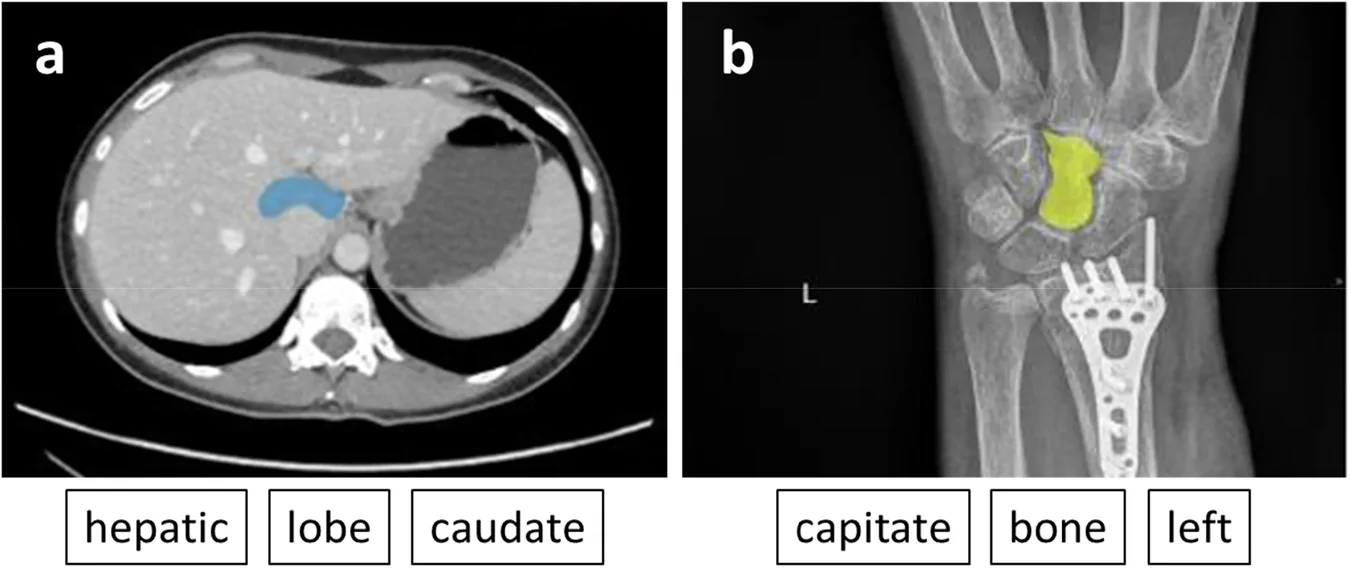
Example images with colored overlaid areas to label the hepatic caudate lobe on an axial CT image (**a**) and the capitate bone on a wrist conventional X-ray (**b**)

The main asset of *ANATOMIADI* centers on a high-fidelity database comprising high-resolution computed tomography (CT), magnetic resonance imaging (MRI), ultrasound (US), and conventional X-ray images. These 2D and 3D datasets were anonymized, archived, and manually labeled by residents of the program of the University of Trento (Fig. 1) to highlight specific anatomical structures, then validated by three senior radiologists (C.C.Q., M.N., A.F.), each with over 20 years of experience. The selection of radiological images was based on the systematic search of items in the analytical index of the treatise “Trattato di Anatomia topografica” by Testut L. and Jacob H. Ed. 2 (ISBN:8802021945). The residents had the freedom to choose any modality and any reformatting plane to show the anatomical structure. The step of validation by senior radiologists ensured pedagogical relevance, specifically for complex anatomical structures and variants. The requirement for written informed consent to use the images was waived because of the retrospective design and the use of anonymized data.

Images were optimized for rapid web-based visualization while maintaining full diagnostic quality. Each entry was indexed in a structured table, linking the image code to up to three descriptors (e.g., ‘right,’ ‘hepatic,’ ‘lobe’ for an axial abdominal CT) (Fig. 1). Descriptive adjectives (e.g., ‘superior,’ ‘anterior’) were included to refine identification. This methodology resulted in the segmentation of over 1800 anatomical structures, associated with more than 4300 descriptors. Structures were categorized into five major regions: (1) Neuro, (2) Head and Neck, (3) Cardio-Thoraco-Vascular, (4) Abdominal & Pelvic, and (5) Musculoskeletal. Overlapping classifications were permitted; for instance, the right meningeal artery at the foramen spinosum was assigned to the Head and Neck, Cardio-Thoraco-Vascular, and Nervous categories. Finally, labeling conventions (filled vs. empty areas) were employed to distinguish between a structure’s content (e.g., a nerve) and its container (e.g., a canal), preventing ambiguity.

### Puzzle development and gamification

To transform traditional anatomical identification into a high-engagement educational challenge, we leveraged the interactive tools provided by the online platform Puzzel.org, founded by Daan Weustenraad (Amersfoort, the Netherlands, https://puzzel.org). Of the 29 activity formats available on Puzzel.org, seven were selected because they could be effectively adapted to image-based anatomy learning. The choice of seven formats was intended to provide sufficient variability in gameplay and cognitive challenges, thereby maintaining participant engagement while avoiding excessive complexity in the design and administration of the competition [9].

**Fig. 2:**
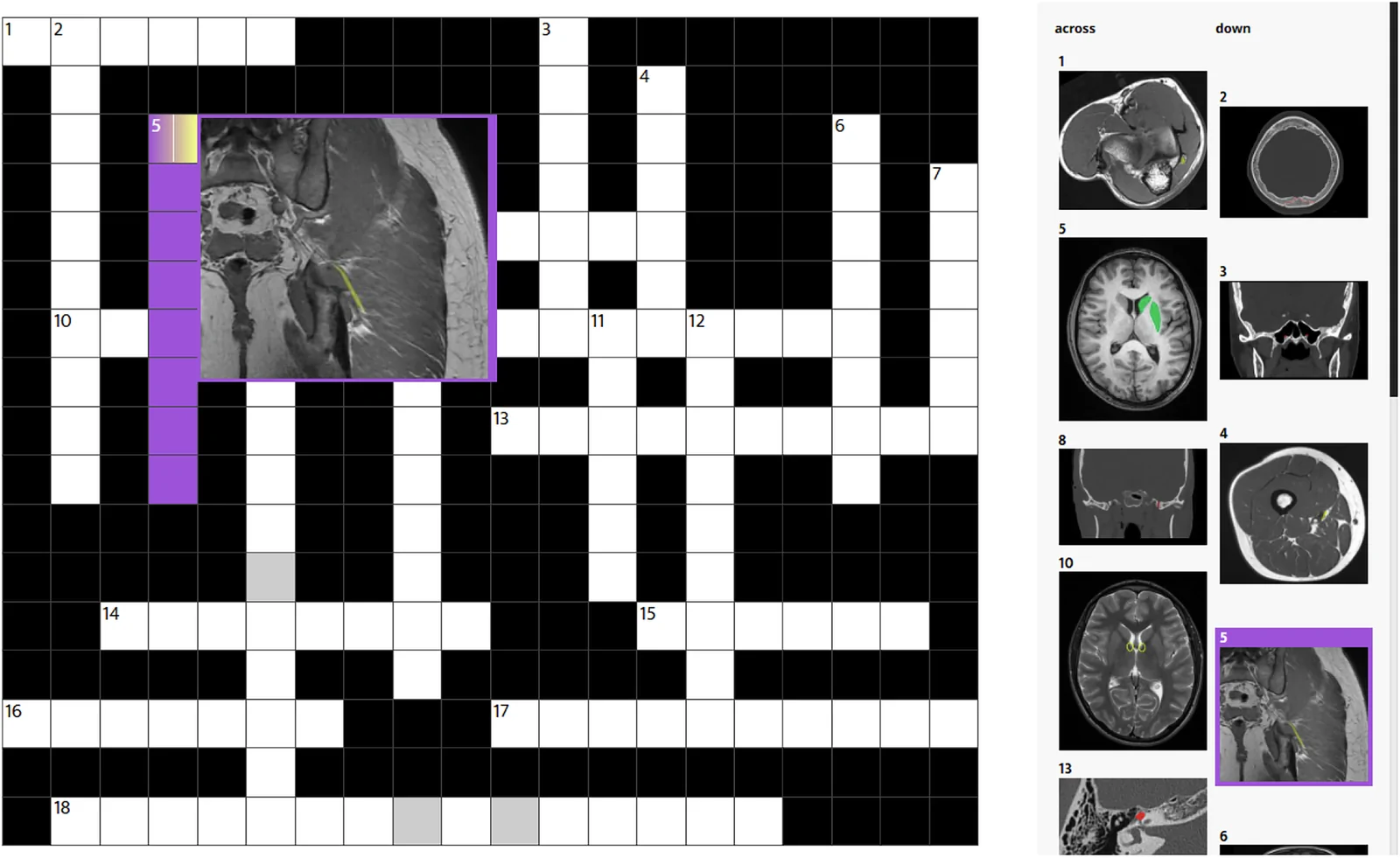
Layout of the crosswords game. A maximum time of 600 s was given to complete it. Single response time was 10 s for the basic level and 15 s for the intermediate and advanced levels of difficulty

This allowed for the development of diverse enigmatics and puzzles, including customized crosswords, drag-and-drop labeling, and visual recognition tasks to evaluate not only precision but also response speed of residents by including tasks to be performed under time constraints. Ultimately, seven game formats were identified as most effective for anatomical instruction: acrostic puzzles, crosswords (Fig. 2), fill-in-the-gap, hangman, keypad tasks, image labeling, and the standard multiple-choice quizzes. Over 400 unique games were developed, categorized by anatomical system and tiered into three levels of difficulty: basic, intermediate, and advanced.

The competition was structured as a head-to-head challenge between two teams, each composed of a maximum of four players (residents). Participants engaged with the same puzzle, with turns alternating after every response, regardless of accuracy. The sessions were conducted via interactive touchscreen interfaces (Samsung Flip Pro Digital Flipboard 65”, Vietnam). The monitor was used to facilitate group-based participation, allowing up to 16 residents to interact simultaneously with a horizontally positioned display and ensuring adequate visibility of image-based content. The large screen was therefore a logistical choice to support collaborative gameplay. For individual use, all activities can be performed on conventional desktop or laptop monitors without loss of functionality; however, this use case was outside the scope of the event.

Faculty members served as moderators/referees, responsible for scoring and ensuring adherence to the specific time constraints established for each game. Additionally, a dedicated staff member was present to provide technical support and manage logistical requirements throughout the exercise.

### Competitive platform and scoring system

The competition was run into two phases: a first round-robin phase on day 1, when teams competed in a league format to establish initial rankings, and a second tournament phase (day 2), when, by a progressive twinning mechanism, losing teams merged with the winners, forming increasingly larger macro-teams. The twinning mechanism consisted of merging the winning and losing teams after each match, with the winning team remaining the leader of the newly formed group. This strategy prevented participant elimination, ensured continuous involvement of all residents throughout the competition, and maintained the competitive hierarchy while fostering collaboration and knowledge sharing. This progressive integration of teams represents the essence of the twinning approach [9]. This collaborative-competitive structure culminated in a Grand Finale between two opposing macro-coalitions, fostering a sense of national community among the participants while maintaining the intensity of the competition. Stringent proctoring procedures were not considered necessary because the competition took place in a supervised, face-to-face setting. Participants were required to switch off their mobile devices, and dedicated referees at each table continuously monitored the activities. This arrangement provided adequate oversight to detect any inappropriate behavior while maintaining the educational and collaborative nature of the event. No episodes of misconduct were identified during the competition. We developed a dedicated digital infrastructure to manage the real-time progression of the two phases. The platform featured a dynamic dashboard for the live display of progressive results and scoring.

**Fig. 3:**
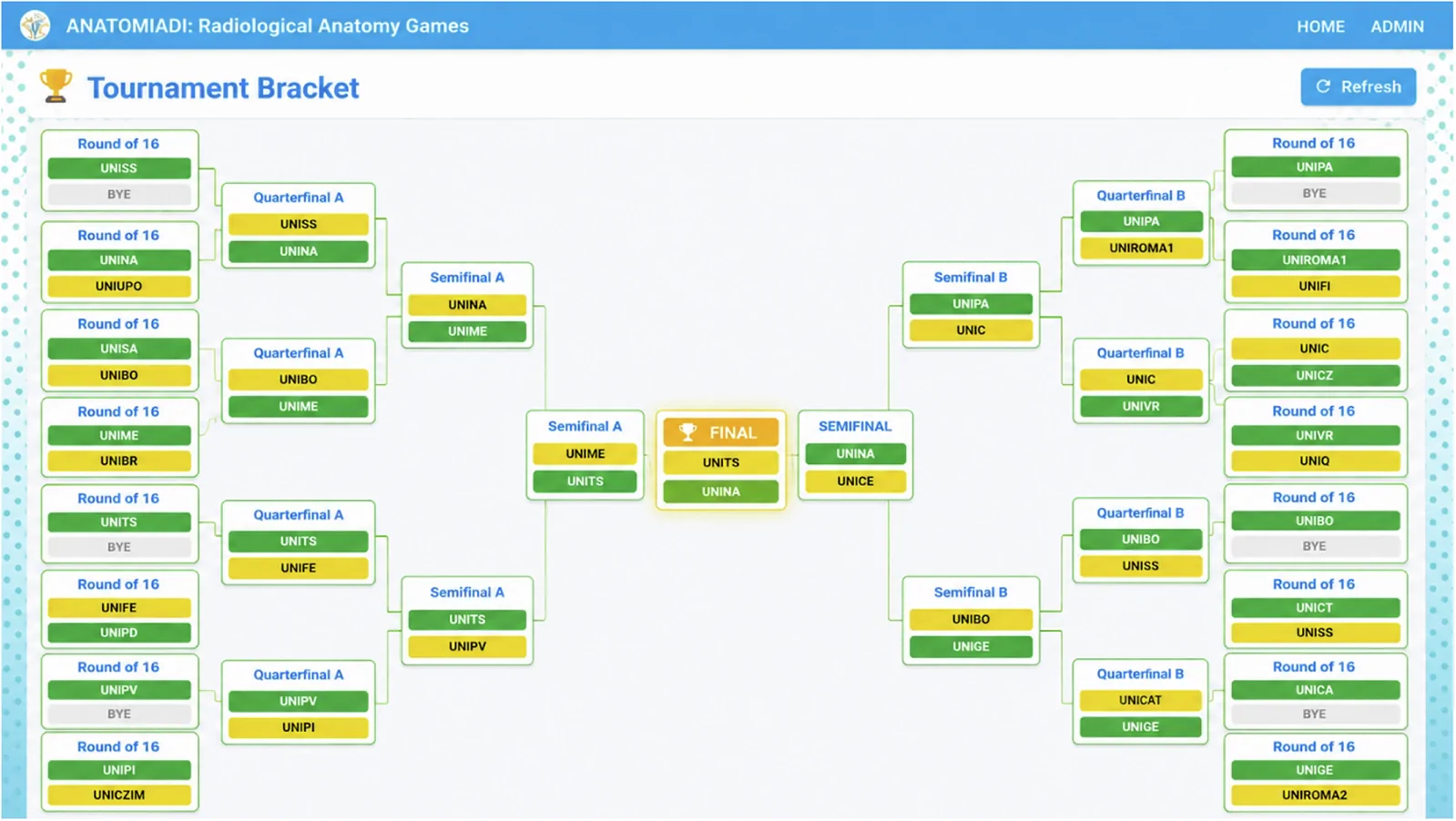
Live display of the league to show the real-time progression during the second day of the *ANATOMIADI* competition

The digital platform developed for the *ANATOMIADI* was built as a web application. The platform is currently developed specifically to support the live event and is taken offline outside the competition period. Therefore, the specific domain is not currently maintained as a permanently accessible public website. Figure 3 shows a representative screenshot used during the competition. The architecture follows a three-tier model: the front-end was developed using Angular, optimized for large screens while remaining responsive for mobile devices; the back-end was based on Node.js with the Express framework; and data persistence was handled by PostgreSQL. To ensure real-time updates during the context, a critical requirement for managing competitive sessions, a WebSocket-based communication layer was integrated. The entire application was containerized using Docker and orchestrated with Docker Compose, adopting a Git-based automated deployment workflow. The infrastructure was hosted on a virtual machine running Ubuntu 22.04 LTS, configured with 4 vCPUs, 8 GB of RAM, and 40 GB of SSD storage, sized to support an estimated load of 50 to 100 concurrent users. Access to the platform was managed through internal authentication, with separate credentials for administrators and referees.

### Feedback survey

As a follow-up to the event, a feedback survey was designed and distributed to the participants of the *ANATOMIADI* including residents, referees/experts, and staff members. The online survey was available to all participants for 30 days after the event and was designed to capture data on different domains regarding the educational value, gamification and engagement, platform usability and team collaboration, technical workflow, logistics, scientific accuracy, fairness, and quality assessment. For most questions, a 5-point Likert scale was used to categorize responses in the different domains.

The survey comprised 30 questions structured into four sections: Section 1—Technical interaction and competition dynamics. Q1, Was the tactile responsiveness of the touchscreen interfaces adequate for the specific requirements of the gamified tasks? Q2, Was the visual layout and User Interface (UI) formatting optimized for large-scale monitor display and participant interaction? Q3, Did the radiological images (CT, MRI, US) maintain sufficient spatial resolution and diagnostic quality to meet the educational objectives? Q4, Were the highlighted anatomical structures clearly discernible within the provided radiological datasets? Q5, Was the distribution of anatomical regions (e.g., nervous, abdomino-pelvic, musculoskeletal) appropriately balanced across the competitive rounds? Q6, Was the progressive increase in question difficulty from Day 1 to Day 2 perceived as appropriate and pedagogically effective? Q7, Were the time limits allocated for each task sufficient to allow for accurate diagnostic reasoning while maintaining competitive pressure? Q8, Was the presence of a dedicated proctor/assistant at each station perceived as helpful for technical support and procedural guidance? Q9, Is the presence of a dedicated assistant considered essential for the successful execution of the game-based tasks?

Section 2—Impact on learning radiological anatomy. Q10, What was the level of knowledge acquired at the end of the event? Q11, What was the level of knowledge required to solve puzzles? Q12, How do you rate the overall contribution to learning radiological anatomy? How do you rate the specific contribution to learning anatomy of the acrostic puzzles (Q13), crosswords (Q14), image labeling (Q15), fill-in-the-gap (Q16), multiple-choice quizzes (Q17), hangman (Q18), keypad (Q19)?

Section 3—Perceived satisfaction and engagement. How do you rate the level of satisfaction/engagement of the acrostic puzzles (Q20), crosswords (Q21), image labeling (Q22), fill-in-the-gap (Q23), multiple-choice quizzes (Q24), hangman (Q25), keypad (Q26)?

Section 4—Peer recommendation and educational advocacy. Q27, open-ended question for general comments about *ANATOMIADI*; Q28, open-ended question to comment on strengths, weaknesses, opportunities, and threats aimed to carry out a SWOT analysis; Q29, Would you recommend a resident in radiology to participate in *ANATOMIADI*? Q30, Which year of residency is the best target for *ANATOMIADI* in the training curriculum (first to fourth year)? (See Appendix 1).

### Statistical analysis

Descriptive statistics were calculated for all survey items assessed with the 1–5 Likert scale. Composite scores were generated for thematic aggregate analysis of survey items (Q1–Q4, Q5, Q6–Q9, Q10–Q12, Q13–Q19, and Q20–Q26). To compare the mean scores across the thematic clusters, a One-Way Analysis of Variance (ANOVA) was conducted. Likert scale data were treated as interval data for the purpose of this analysis. The assumption of homogeneity of variances was assessed using Levene’s Test, and post hoc comparisons were performed using Tukey’s HSD (Honestly Significant Difference) to identify specific pairwise differences between groups. Statistical significance was set at *p* < 0.05.

## Results

*ANATOMIADI* was held in Rovereto (Trento, Italy) on December 11–12, 2025, with an attendance of 141 participants representing 28 residency programs across Italy. The cohort included 98 residents, supported by 29 referees and 14 staff members. A total of 63 (44.7%) participants completed the feedback survey within 1 month after the event. Respondents represented, proportionally balanced, the three different groups, with the majority being residents (74.6%, 47/63), followed by referees (15.9%, 10/63) and staff members (9.5%, 6/63). Survey results are summarized in Table 1.

**Table 1:** Survey results presented in terms of agreement (positive)

Questions	Disagree	Neutral	Agree	% Positive	Mean	SD
Q1	11	12	40	63.5	3.49	1.01
Q2	16	17	30	47.6	3.27	1.11
Q3	5	3	55	87.3	4.19	1.01
Q4	4	11	48	76.2	3.90	0.91
Q5	18	10	35	55.6	3.37	1.18
Q6	2	8	53	84.1	4.24	0.93
Q7	15	13	35	55.6	3.41	1.14
Q8	6	9	48	76.2	4.13	1.07
Q9	3	2	58	92.1	4.48	0.88
Q10	6	20	37	58.7	3.63	0.99
Q11	4	8	51	81.0	4.10	0.90
Q12	8	14	41	65.1	3.73	1.10
Q13	5	20	38	60.3	3.67	0.97
Q14	3	9	51	81.0	4.06	0.86
Q15	5	15	43	68.3	3.84	1.02
Q16	5	14	44	69.8	3.95	1.01
Q17	9	11	43	68.3	3.67	1.09
Q18	7	13	43	68.3	3.78	0.96
Q19	8	12	43	68.3	3.86	1.11
Q20	5	11	47	74.6	3.92	0.94
Q21	1	6	56	88.9	4.48	0.69
Q22	5	16	42	66.7	3.81	0.98
Q23	15	16	32	50.8	3.30	1.19
Q24	11	12	40	63.5	3.65	1.18
Q25	10	7	46	73.0	3.87	1.22
Q26	13	12	38	60.3	3.68	1.28

Disagree = Likert Scale 1 and 2; Neutral = Likert Scale 3; Agree = Likert Scale 4 and 5

*SD* standard deviation

The survey results indicated a generally positive perception across most items. The highest level of agreement was observed for Q9, where 92.1% of participants reported positive responses (mean ± SD = 4.48 ± 0.88). Similarly, Q3 and Q6 showed strong consensus with 87.3% and 84.1% (mean ± SD = 4.24 ± 0.93) agreement, respectively.

Conversely, Q2 was the most divisive item, yielding the lowest agreement rate (47.6%), the lowest mean score (mean ± SD = 3.27 ± 1.11), and the highest frequency of neutral responses (17.3%). Items Q5 and Q7 also showed higher levels of disagreement compared to the rest of the respondents, with approximately 28% and 24% of respondents expressing negative views.

Notably, Q5 and Q7 exhibited the highest variance (1.18 and 1.14, respectively), suggesting a lack of consensus among the sample.

The one-way ANOVA revealed a significant effect of thematic grouping on participant responses (*p* < 0.001). Post hoc comparisons using the Tukey HSD test indicated that the mean score for Q6-Q9 (mean ± SD = 4.07 ± 1.00) was significantly higher than Q1-Q4 (mean ± SD = 3.71 ± 1.01, *p* = 0.01) and Q5 (mean ± SD = 3.37 ± 1.18, *p* < 0.001). No statistically significant difference was found between Q1-Q4 and Q5, suggesting that the assistance of the dedicated staff was crucial for the perceived success of interaction with touchscreens.

The descriptive analysis of items Q10 through Q19 revealed a consistently positive trend, with all mean scores exceeding the theoretical midpoint of 3.0. The highest levels of agreement (51/63 positive responses) were recorded for Q11 (mean ± SD = 4.10 ± 0.90) and Q14 (mean ± SD = 4.06 ± 0.86). Q10 exhibited the lowest mean score (mean ± SD = 3.63 ± 0.99). Q10 and Q13 received the highest number of neutral responses (20 out of 63, 31.7%). Q17 received the highest number of negative responses (9 out of 63, 14.3%).

Analysis of items Q20-Q26 revealed that Q21 reached the highest level of consensus (mean ± SD = 4.48 ± 0.69) with 88.9% of respondents reporting positive scores on crosswords. Q23 showed fill-in-the-gap as the least favored puzzle (mean ± SD = 3.30 ± 1.19), showing an agreement rate of only 50.8% and the highest number of disagreements (*n* = 15). Q26 (keypad) exhibited the highest variance (mean ± SD = 3.68 ± 1.28), suggesting a conflictual perception among participants.

Open-ended feedback (Q27) revealed an overwhelmingly positive reception of the *ANATOMIADI* competition. The comments could be synthesized into four major themes: (1) innovation and game-based format as a powerful stimulus to identify individual knowledge gaps and to drive a rigorous study of anatomy; (2) networking at national level as the competition provided a framework to connect and compare different levels of expertise; (3) engagement and motivation as many comments praised the value of “healthy competition” and “team building”; (4) inclusivity of the event as it is uncommon to provide such an experience in postgraduate training. While some technical touchscreen issues and procedural ambiguities were noted, they did not diminish the overall “extremely positive judgment” of the initiative.

**Fig. 4:**
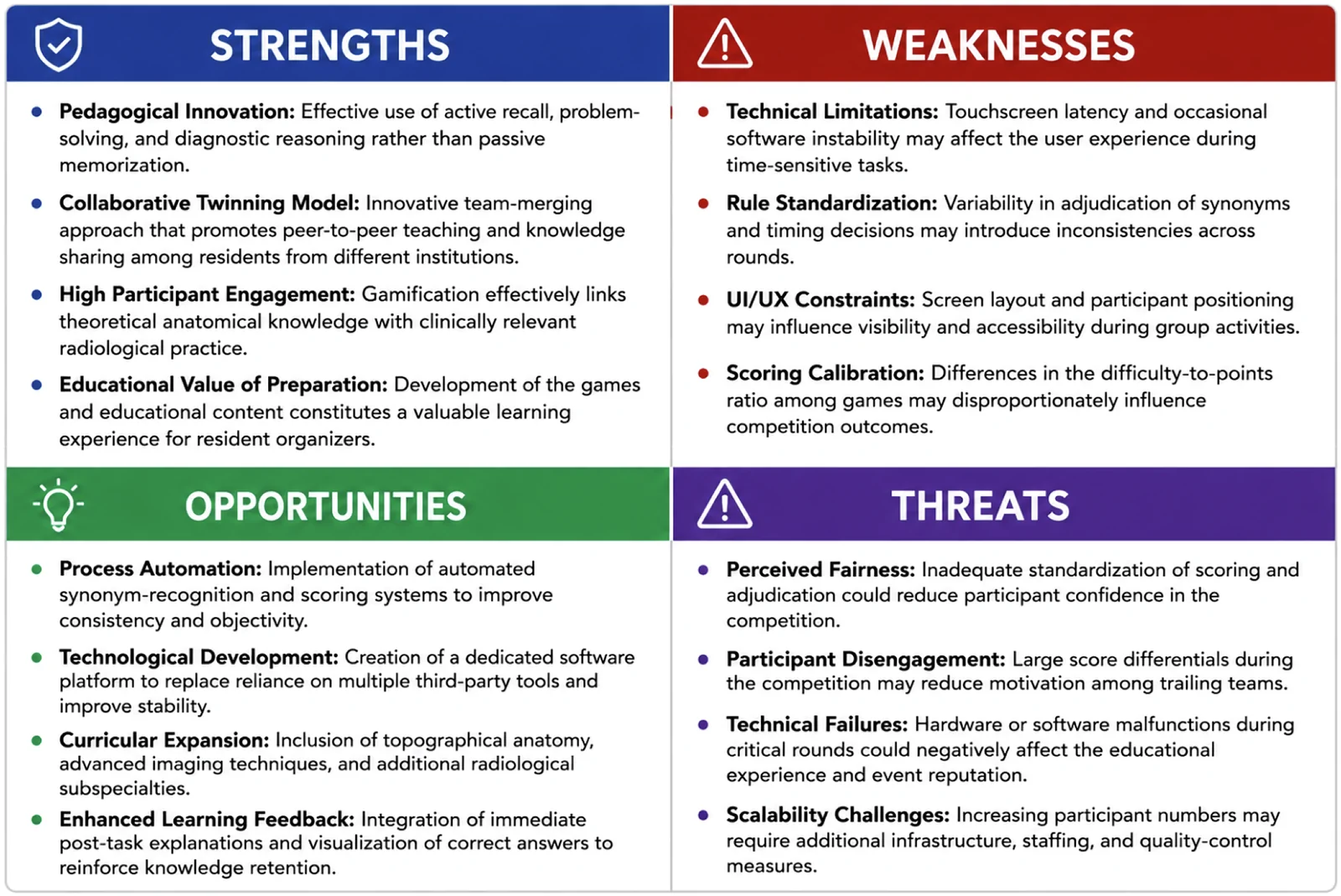
SWOT analysis summarizing responses to open-ended Q28

Responses to Q28 allowed us to build a SWOT analysis presented in Fig. 4.

The evaluation of the event’s perceived value yielded a unanimous positive response; indeed, 100% of the participants recommended that the competition be repeated in future academic years (Q29).

Responses to Q30 showed that, based on the required level of knowledge, the event would be recommended to residents in their 3rd (54 out of 63 responses) and 4th year (48 out of 63 responses) of postgraduate education. In fact, only 7 respondents recommended the event for 1st-year residents. This distribution also suggested that the competition’s complexity and collaborative elements can be particularly well-received by those in more advanced stages of training.

## Discussion

The results of the feedback survey on the *ANATOMIADI* competition demonstrate that gamification is a powerful pedagogical tool for radiological anatomy, significantly enhancing engagement and peer-to-peer knowledge transfer among residents [10–12]. The high level of satisfaction and the unanimous recommendation for future editions (100% agreement among respondents) underscore the demand for innovative, interactive formats in postgraduate medical education. In fact, while gamification of anatomy has demonstrated to increase learner engagement, motivation, and knowledge retention in medical graduate training [2, 13], its role in postgraduate radiology training has been frequently undervalued, and a high level of anatomical proficiency is a critical determinant of diagnostic accuracy and clinical decision-making.

A key finding of this study was the critical role of human-mediated technical support. While participants reported high satisfaction with the digital interfaces and image quality, the significant divergence in opinions regarding the large-scale UI suggests that the physical layout of the competition workstations required manual intervention from proctors to ensure a smooth experience. The presence of dedicated assistants was perceived as a more effective feature than the standalone technical interaction, highlighting that in high-pressure gamified environments, technical reliability must be paired with immediate human troubleshooting. In addition, these results support the idea that efforts to adapt the environment to learners’ specific needs can increase motivation and engagement [14].

Pedagogically, the twinning mechanism transformed a standard competition into a collaborative learning ecosystem. By merging teams, the event facilitated inter-institutional benchmarking, allowing residents from 28 different programs to compare expertise and bridge knowledge gaps through active recall. The preference for crosswords over more traditional formats like multiple-choice quizzes or fill-in-the-gap tasks suggests that puzzles requiring constructive retrieval of anatomical terms are more effective for long-term retention than recognition-based tasks.

Furthermore, the data regarding the target audience indicated that the competition’s complexity is best suited for senior residents (3rd and 4th years). In fact, the competition was designed to test and reinforce existing knowledge of radiological anatomy rather than to teach basic anatomy. Therefore, the challenges were intentionally structured with progressively increasing levels of difficulty, requiring participants to recognize anatomical structures across different imaging planes, contrast settings, and reformatted images. While the team-based format facilitated collaborative problem solving, the complexity of the tasks was such that junior residents (particularly those in their first or second year of training) may not have had sufficient experience to fully engage with all challenges independently.

Despite the positive outcomes, several limitations must be acknowledged. First, the sample size of survey respondents (*n* = 63) represents only 44.6% of the total participants, which may introduce a self-selection bias toward those who had a more positive experience. Second, the technical disparities noted in touchscreen responsiveness and the lack of a standardized synonym-recognition engine for anatomical terms (e.g., Latin vs. Italian nomenclature) may have introduced “noise” into the scoring system, potentially affecting the perceived fairness of certain rounds. Third, reproducibility on standard displays was not tested and remains unverified, but it will be explored in future studies. Fourth, the cross-sectional nature of the survey measures “perceived” learning and satisfaction rather than objective long-term knowledge retention. While participants felt they acquired significant knowledge, a follow-up assessment would be required to quantify the actual educational impact. In this regard, the event is intended to become an annual initiative, progressively implementing additional tools and metrics to evaluate its educational effectiveness. This iterative process will help establish the extent to which the approach enhances learning outcomes and determine its relative value compared with traditional educational methods in radiology training. Finally, the logistical challenges of managing large “macro-teams” in the final rounds may have diluted individual participation, suggesting that future iterations should refine the collaborative workflow to ensure equitable engagement for all team members.

The *ANATOMIADI* competition may represent a paradigm shift in postgraduate radiological education, successfully bridging the gap between theoretical anatomical knowledge and practical diagnostic proficiency through gamification. The post-event analysis demonstrates that a multi-institutional, competitive-collaborative framework significantly enhances learner engagement, accelerates the mastery of complex 3D structures, and fosters a robust national professional network among residents. While the pilot edition highlighted areas for technical refinement—specifically regarding UI/UX optimization, automated synonym recognition, and the standardization of refereeing protocols—the overwhelming peer-to-peer recommendation rate validates the model’s effectiveness. Based on open-ended responses, the “progressive twinning” mechanism was perceived by participants as a powerful tool transforming individual competition into a collective educational experience. The 100% recommendation rate was particularly notable given the diverse institutional backgrounds of the residents. This suggests that the benefits of the competition—specifically active recall and inter-institutional benchmarking—transcend local curricular differences, making it a universally applicable educational tool.

In conclusion, the *ANATOMIADI* format offers a scalable and reproducible blueprint for modernizing pedagogy in radiology. Integrating high-fidelity imaging with interactive game mechanics builds the foundation for a more collaborative and technologically adept generation of radiologists. Future iterations, incorporating the feedback-driven improvements identified in this study and eventually empowerment through artificial intelligence [15], have the potential to set a new gold standard for radiological training across Europe.

## Supplementary information


ELECTRONIC SUPPLEMENTARY MATERIAL


## Data Availability

Data used for analysis can be made available to interested parties upon request.

## Abbreviations

ANOVA: One-way analysis of variance
CT: Computed tomography
GBL: Game-based learning
MRI: Magnetic resonance imaging
UI: User interface
US: Ultrasound
