# Supplementary material for: ANATOMIADI: a national, image-based anatomy competition for residents in Italy to enhance postgraduate education through interactive learning
Source: Insights Imaging. 2026 Aug 29;17:215. doi: 10.1186/s13244-026-02389-z (PMC13525974; doi:10.1186/s13244-026-02389-z)
Supplement: Supplementary file 1 [file 13244_2026_2389_MOESM1_ESM.pdf]

ANATOMIADI: A national, image-based anatomy competition for residents in Italy to enhance postgraduate education through interactive learning.

ELECTRONIC SUPPLEMENTARY MATERIAL

Appendix 1.

ANATOMIADI – Participant Feedback Survey

Scale for Q1–Q26:

1=Strongly Disagree/Very Low, 2=Disagree/Low, 3=Neutral, 4=Agree/High, 5=Strongly Agree/Very High.

Question	1	2	3	4	5
**Q1** Touchscreen responsiveness was adequate	☐	☐	☐	☐	☐
**Q2** UI was optimized for large monitor interaction	☐	☐	☐	☐	☐
**Q3** Image quality was sufficient	☐	☐	☐	☐	☐
**Q4** Structures were clearly discernible	☐	☐	☐	☐	☐
**Q5** Anatomical regions were appropriately balanced	☐	☐	☐	☐	☐
**Q6** Progressive difficulty was appropriate	☐	☐	☐	☐	☐
**Q7** Time constraints were adequate	☐	☐	☐	☐	☐
**Q8** Assistant/proctor was helpful	☐	☐	☐	☐	☐
**Q9** Assistant/proctor was essential	☐	☐	☐	☐	☐
**Q10** Level of knowledge acquired	☐	☐	☐	☐	☐
**Q11** Level of knowledge required was appropriate	☐	☐	☐	☐	☐
**Q12** Overall contribution to learning anatomy	☐	☐	☐	☐	☐
**Q13** Specific contribution to learning: Acrostic	☐	☐	☐	☐	☐
**Q14** Specific contribution to learning: Crossword	☐	☐	☐	☐	☐
**Q15** Specific contribution to learning: Image-labeling	☐	☐	☐	☐	☐
**Q16** Specific contribution to learning: Fill-in-the-gap	☐	☐	☐	☐	☐
**Q17** Specific contribution to learning: Multiple-choice	☐	☐	☐	☐	☐
**Q18** Specific contribution to learning: Hangman	☐	☐	☐	☐	☐
**Q19** Specific contribution to learning: Keypad	☐	☐	☐	☐	☐
**Q20** Level of satisfaction/engagement: Acrostic	☐	☐	☐	☐	☐
**Q21** Level of satisfaction/engagement: Crossword	☐	☐	☐	☐	☐
**Q22** Level of satisfaction/engagement: Image-labeling	☐	☐	☐	☐	☐
**Q23** Level of satisfaction/engagement: Fill-in-the-gap	☐	☐	☐	☐	☐
**Q24** Level of satisfaction/engagement: Multiple-choice	☐	☐	☐	☐	☐
**Q25** Level of satisfaction/engagement: Hangman	☐	☐	☐	☐	☐
**Q26** Level of satisfaction/engagement: Keypad	☐	☐	☐	☐	☐

Q27 General comments:

____

____

Q28 SWOT:
Strenghts:____
Weaknesses:____
Opportunities:____
Threats:____

Q29 Would you recommend Anatomiadi to a radiology resident? ☐ Yes ☐ No

Q30 Which is the best target residency year for Anatomiadi? ☐ 1st ☐ 2nd ☐ 3rd ☐ 4th
